# Ortho-Topolin Riboside Induced Differentiation through Inhibition of STAT3 Signaling in Acute Myeloid Leukemia HL-60 Cells

**DOI:** 10.4274/tjh.galenos.2019.2019.0020

**Published:** 2019-08-02

**Authors:** Li Wang, Jiao Cheng, FanLin Lin, ShengXian Liu, Hui Pan, MingDa Li, ShanShan Li, Na Li, WeiPing Li

**Affiliations:** 1 School of Life and Medicine, Dalian University of Technology, PanJin, China; 2 The Second Hospital of Dalian Medical University, Dalian, China

**Keywords:** Ortho-topolin riboside, Differentiation, STAT3 signal, HL-60 cells

## Abstract

**Objective:**

We previously demonstrated that ortho-topolin riboside (oTR) as a naturally occurring cytokinin secreted from *Populus* × *robusta* has great potential anticancer effects via the mitochondrial apoptotic pathway and endoplasmic reticulum stress pathway. In the present study, we reveal that oTR induced the differentiation of acute myeloid leukemia (AML) HL-60 cells, which represent the M2 subtype of AML.

**Materials and Methods:**

After the incubation of HL-60 cells with oTR, its effect was analyzed with cell viability assay, Wright-Giemsa staining, CD11b protein expression analysis, western blot analysis, and polymerase chain reaction.

**Results:**

We found that oTR arrested the cell cycle at the S phase, upregulated the expression of myeloid surface marker CD11b, reduced the nuclear cytoplasmic ratio, and altered the horseshoe shape of nuclei, as evidenced by Wright-Giemsa staining. Furthermore, we found that the protein level of phosphorylated STAT3 was decreased when cells were treated with oTR, while phosphorylated STAT1 was activated. Moreover, the protein level of phosphorylated STAT3 and its upstream kinase, Janus kinase 2, were also inhibited when cells were treated with oTR after increased time. Additionally, the levels of phosphorylated SHP-1 were increased while phosphorylated SHP-2 was decreased.

**Conclusion:**

Collectively, our data indicate a differentiation-induced mechanism underlying the inhibition of STAT3 signaling upon treatment with oTR. Therefore, oTR may constitute a novel differentiation-induced therapeutic for use in clinical treatment of AML.

## Abstract (tr)

**Amaç:**

Daha önceki çalışmalarımızda *Populus* × *robusta*’dan doğal olarak oluşan sitokin orto-topolin ribozidin (oTR) mitokondrial apopitotik yolaklar ve endoplazmik retikulum stres yolakları vasıtasıyla önemli bir antikanser potansiyelinin olduğunu göstermiştik. Bu çalışmada, oTR’nin AML M2 subtipi özelliğindeki HL-60 hücre serisinde diferansiyasyonu indüklediğini gösterdik.

**Gereç ve Yöntemler:**

HL-60 hücrelerinin oTR ile inkübasyonunu takiben hücre üzerideki etkileri hücre canlılık testleri, Wright-Giemsa boyaması, CD11b protein ekspresyon analizi, western blot analizi ve polimeraz zincir reaksiyonu ile araştırıldı.

**Bulgular:**

oTR’nin hücre siklusunun S fazında duraklattığını, myeloid hücre yüzey belirteçlerinden CD11b ekspresyonunun arttığını, çekirdek sitoplazma oranının azaldığını ve çekirdeğin atnalı şeklinin değiştiğini Wright-Giemsa boyası ile destekleyerek gördük. oTR ile muamele edilmiş hücrelerde fosforile STAT3 protein düzeyinin azaldığını, fosforile STAT1’in ise aktive olduğunu bulduk. Ayrıca fosforile STAT3 ve yukarı yöndeki kinaz olan Janus kinaz 2’nin, hücrelerin oTR ile inkübasyonunda artmış zamanla inhibe olduğu görüldü. Ek olarak fosforile SHP-1 düzeyleri artarken fosforile SHP-2 düzeyi azaldı.

**Sonuç:**

Birlikte değerlendirildiğinde, sonuçlarımız oTR ile STAT3 inhibisyonu üzerinden bir diferansiyasyon indükleme mekanizmasını işaret etmektedir. Bu nedenle, oTR AML tedavisinde yeni bir diferansiyasyon-indükleyici terapötik olarak yer alabilir.

## Introduction

Leukemia comprises a group of malignant blood diseases characterized by uncontrolled overproduction of hematopoietic progenitors or terminally differentiated leukocytes [1]. It remains particularly difficult to treat acute myeloid leukemia (AML) [2]. Thus far, cytotoxic drugs targeting proliferating cells have shown limited efficacy in the treatment of AML; notably, such drugs also exhibit significant toxicity. All-trans retinoic acid (ATRA) and arsenic trioxide (ATO) provide new options for differentiation therapy, but have been limited to treatments of AML-M3 and are not suitable for other subtypes of AML [3]. Therefore, development of new chemotherapy drugs that can effectively promote differentiation and eliminate AML is urgently needed.

The level of tyrosine phosphorylation must be balanced during a variety of cellular processes such as growth and differentiation. This is maintained by protein tyrosine kinases and protein tyrosine phosphatases (PTPs) [4]. SHP-1 and SHP-2 (SH2 domain-containing phosphatases 1 and 2) are two PTPs that play important roles in lymphocytes and other hematopoietic cells [5]. Signal transducer and activator of transcription (STAT) proteins are very important in the regulation of cell proliferation, survival, differentiation, and immune response [6]. It has been reported that aberrant STAT signaling often occurred in cases of AML [7,8,9]. Notably, some natural products have been reported to inhibit STAT3 activity through the regulation of SHP-1 and/or SHP-2 in cancer cells [10].

Cytokinins are important phytohormones that control a variety of cellular processes in plants [11]. Moreover, cytokinin ribosides (N6-substituted adenosines) exhibit significant anticancer activity in mammals [12,13]. It was found that cytokinin ribosides can induce cell apoptosis and block cell cycling in various cancer cell lines, as well as in several xenografts and in a small clinical trial [14].

**Figure 1:**
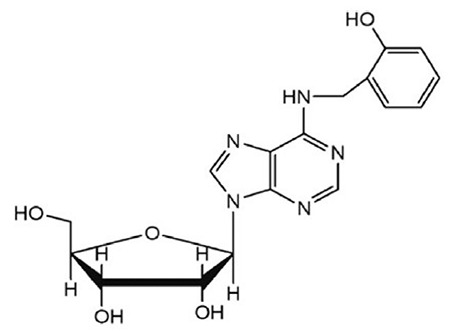
Structure of ortho-topolin riboside.

Ortho-topolin riboside (oTR; also known as 6-(2-hydroxybenzylamino)-9-D-ribofuranosylpurine, Figure 1) is a naturally occurring cytokinin secreted from *Populus* × *robusta* leaves after sunrise [15]. oTR has shown unique cytotoxic activity against NCI_60_ cell lines compared with the activity of other cytokinin ribosides [14]. However, a detailed molecular mechanism underlying the effect of oTR on differentiation has not been elucidated with respect to AML cells. We previously reported that oTR exhibited antitumor activity by inducing differentiation in the U937 human leukemia cell line, but the differentiation-inducing properties of oTR remain undefined in HL-60 cells. In this study, we detected the antitumor effect of oTR on HL-60 cells.

## Materials and Methods

### Materials

Ortho-topolin riboside (oTR, purity >99%) was obtained from OlChemim GmbH (Czech Republic). RPMI 1640 and fetal bovine serum (FBS) were obtained from GIBCO (USA). Anti-STAT3, anti-phospho-STAT3^Y705^, anti-JAK2, anti-phospho-JAK2^Y1007/1008^, anti-phospho-SHP-1^Tyr564^, anti-phospho-SHP-2^Tyr542^, anti-SHP-1, anti-SHP-2, and β-actin were obtained from Cell Signaling (USA). Wright-Giemsa staining solution was obtained from Sigma-Aldrich Corporation (USA). Penicillin-streptomycin, Cell Counting Kit-8 (CCK-8), and phosphatase inhibitor complex were obtained from the Beyotime Institute of Biotechnology (Beijing, China). Anti-human CD11b-PE was obtained from eBioscience (USA).

### Cell Culture

HL-60 cells were obtained from the Cell Bank of the Chinese Academy of Sciences (Shanghai, China). Cells were incubated in complete RPMI 1640 (RPMI 1640 with 10% (v/v) FBS and 100 U/mL of penicillin and streptomycin) at 37 °C in a humidified atmosphere containing 5% CO_2_.

### Cell Viability Assay

Cell viability was assayed using the CCK-8 assay. At a density of 1x10^4^ cells/100 µL per well the cells were seeded, and then they were treated with increasing concentrations of oTR (0.1, 1, 10, 50, and 100 µM). After 24 h incubation, 10 µL of the CCK-8 solution was added to each well for a further 3 h of incubation at 37 °C. Then the absorbance was detected at 450 nm using a microplate reader (3001, Thermo Scientific, Finland). The cell viability was expressed as sample OD/control OD x 100%.

### Wright-Giemsa Staining

After treating the cells in the 96-well plate with the compound for different lengths of time, the cells were collected and washed with PBS. Cells were mounted on glass slides by bench-top low speed centrifuge (L2-4K, Hunan, China), and morphological evaluation of differentiation was assessed using a Wright-Giemsa staining kit. Samples were dried overnight at room temperature and observed using a microscope (DMI 4000B, Leica, Germany).

### CD11b Protein Expression Analysis

The treated cells were collected and analyzed for expression of the cell surface differentiation marker CD11b using a FACSCalibur flow cytometer (FACSCalibur, Becton Dickinson, USA). After treatment, cells were harvested and washed with PBS, and the cells were incubated with the blocking antibody anti-mouse CD16/CD32 for 15 min at room temperature, then incubated with anti-human CD11b-PE antibody for 30 min at room temperature in the dark, and then analyzed by flow cytometry.

### Western Blot Analysis

After treatment with oTR for the time indicated, cells were washed twice with PBS buffer and then resuspended in RIPA buffer. After incubation on ice for 20 min, the RIPA buffer was centrifuged at 12,000 rpm for 20 min at 4 °C. The protein concentrations were measured by Bio-Rad protein assay (Bio-Rad, USA). The protein extracts (30 µg) were boiled and resolved by 12% sodium dodecyl sulfate polyacrylamide gel electrophoresis. After transfer to polyvinylidene fluoride membranes, the membranes were blocked with 5% dry milk in Tris-buffered saline Tween-20 (TBST) at room temperature for 1 h. The membrane was probed with the indicated primary antibody for STAT3, phospho-STAT3, JAK2, phospho-JAK2, SHP-1, SHP-2, phospho-SHP-1, phospho-SHP-2, and β-actin and then incubated with horseradish peroxidase-conjugated secondary antibody for 1 h. Immunoreactive proteins were detected by a chemiluminescence blotting detection system (FluorChem HD2, Alpha Innotech, USA).

### Reverse Transcription-Polymerase Chain Reaction Analysis

The oTR-treated HL-60 cells were collected and washed with PBS buffer, and the total RNA was extracted with TRIzol according to the reagent instructions and quantified by NanoDrop. According to the manufacturer’s instructions, the genomic DNA was removed by adding gDNA Eraser Buffer, gDNA Eraser, total RNA, and RNase-free dH_2_O using the PrimeScriptRT kit. Then the above DNA-removing reaction solution, which was mixed with RNase-free dH_2_O, PrimeScript Buffer, RT Prime Mix, and PrimeScript RT Enzyme Mix, was reverse-transcribed using a Thermal Cycler Dice instrument (TaKaRa, Japan). The polymerase chain reaction (PCR) mixture consisted of TB Green Premix Ex Taq II (Tli RNaseH Plus), PCR primer, DNA template, and sterilized ddH_2_O according to the kit (TaKaRa, No. RR820A). The cycle threshold (CT) value of each gene mRNA was detected by real-time PCR using the LightCycler 96 and analyzed by 2^-ΔΔCT^ method. The PCR primer sequences are given in Table 1.

### Statistical Analysis

For the RT-PCR analysis, the CT was determined using the default settings: ΔCT(test) = CT(target gene, test) - CT(GAPDH, test); ΔCT(calibrator) = CT(target gene, calibrator) - CT(GAPDH, calibrator); ΔΔCT = ΔCT(test) - ΔCT(calibrator); Relative gene expression ratio = 2^-^^ΔΔ^^CT^.

Data are expressed as mean ± standard deviation. Student’s t-test and one-way ANOVA were used to compare the test and control values. *p<0.05 and **p<0.01 were considered statistically significant compared to the control.

## Results

### oTR Inhibited Cell Proliferation in HL-60 Cells

**Figure 2:**
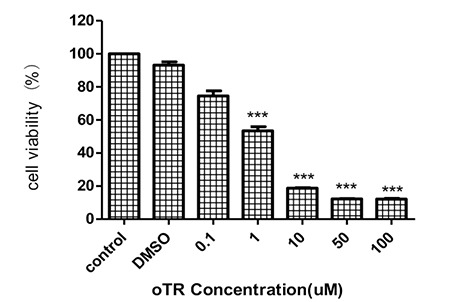
Effects of cell viability of ortho-topolin riboside (oTR) on HL-60 cells. Cell viability was analyzed by CCK-8 assay after 24 h of treatment with increasing concentrations of oTR. The values are expressed as mean ± standard deviation from three individual experiments. ***p<0.001 versus control.

After treatment with increasing concentrations of oTR (0.1, 1, 10, 50, and 100 µM) for 24 h in HL-60 cells, we tested the cell proliferation by the CCK-8 assay. The results showed that the cell viability was inhibited significantly from 91.1±1.3% to 11.3±2.1% upon treatment with increased concentrations of oTR. The half maximal inhibitory concentration (IC_50_)value was 3.4 µM (Figure 2).

### oTR Induced the Differentiation of HL-60 cells

**Figure 3:**
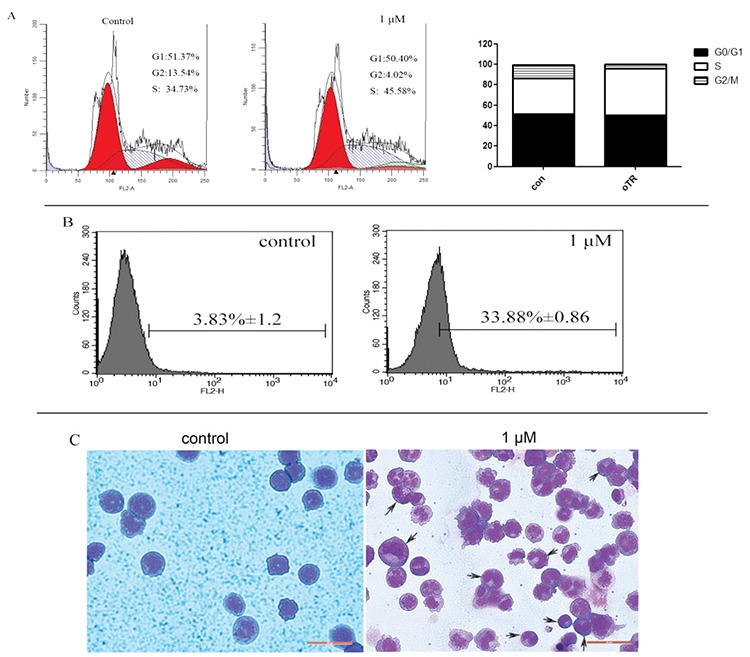
Differentiation-inducing activity of ortho-topolin riboside (oTR) in HL-60 cells. A) The HL-60 cells were treated with oTR (1 μM) for 24 h, and cell cycle analysis was detected by flow cytometry. B) The percentage of CD11b expressed in HL-60 cells. *p<0.05, **p<0.01 versus the control group without any treatment. C) Effects of oTR on the morphology of HL-60 cells. Cells were treated with 1 μM oTR or vehicle (0.1% DMSO) as a positive control for 24 h, and morphological changes were observed by phase contrast microscopy. Black arrows: Differentiated cells.

To detect antiproliferative activity in HL-60 cells, the changes in cell cycle arrest were examined upon treatment with oTR. We found that oTR arrested the cell cycle at S in HL-60 cells (Figure 3A). Because cell growth arrest is coupled with cell differentiation in cancer cells, we used CD11b as a mature granulocyte marker to test the differentiation of HL-60 cells. We found that the levels of the myeloid CD11b marker were elevated upon treatment with 1 µM oTR for 24 h (Figure 3B). We also confirmed induced differentiation by morphological analysis using Wright-Giemsa staining. We found that untreated HL-60 cells were round with large and round nuclei and sparse cytoplasm. Treatment with oTR reduced the nuclear cytoplasmic ratio and altered the horseshoe morphology of nuclei (Figure 3C). All these findings indicated that oTR induced the differentiation of HL-60 cells into mature granulocytes.

### oTR Suppressed STAT3 Activation in oTR-induced AML Differentiation While Inducing the Activation of STAT1

**Figure 4:**
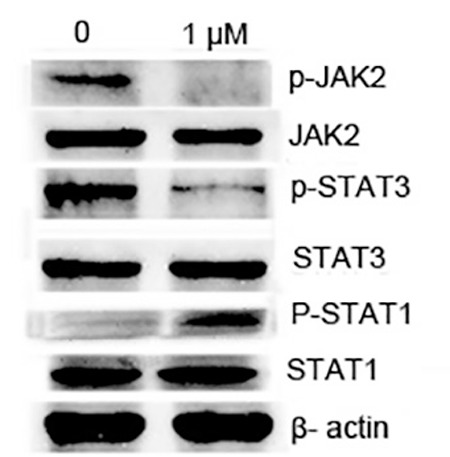
Effects of ortho-topolin riboside (oTR) on protein levels of phosphorylated STAT1, STAT3, and JAK in HL-60 cells. HL-60 cells were treated with oTR (1 μM) for 24 h. The western blot experiments were repeated three times and the data show representative results.

It has been reported that STAT proteins, as latent cytoplasmic transcription factors,  are very important in cellular processes including cell proliferation, differentiation, and apoptosis [16,17]. Here we found that oTR inhibited the protein levels of p-JAK2^Tyr1007^ and p-STAT3^tyr705^ obviously, but not the total protein of JAK2 and STAT3 in HL-60 cells (Figure 4). We also determined whether the activation of STAT1 phosphorylation is involved in the differentiation. Here we found that oTR induced differentiation in HL-60 cells by acting through the activation of p-STAT1.

### oTR Changed the Effects of oTR on mRNA Levels of C/EBPα, C/EBPβ, and PU.1

**Figure 5:**
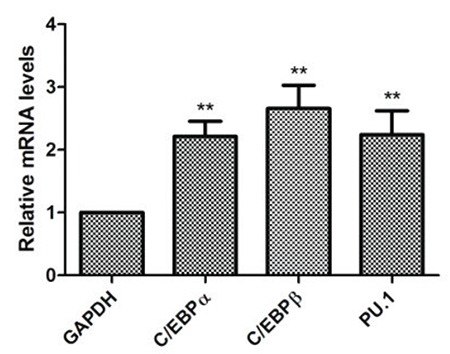
Cells were treated with 1 μM oTR for 48 h. The mRNA expressions of C/EBPα, C/EBPβ, and PU.1 were detected by qRT-PCR. Data presented are the mean ± standard deviation of three independent experiments. **p<0.01.

To further examine the ability of myeloid differentiation after treatment with oTR in HL-60 cells, we detected the mRNA expression of the monocytic transcription factors C/EBPα, C/EBPβ, and PU.1. Consistent with monocytic differentiation, we found that C/EBPα, C/EBPβ, and PU.1 were upregulated compared with untreated cells at 48 h after treatment with oTR (Figure 5).

### oTR Decreased the Phosphorylation of SHP-2 While Increasing the Phosphorylation of SHP-1

**Figure 6:**
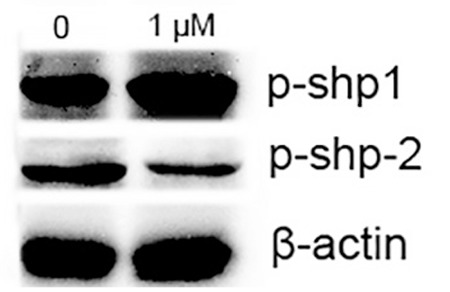
Effects of ortho-topolin riboside (oTR) on protein levels of phosphorylated SHP-1 and SHP-2 in HL-60 cells. HL-60 cells were treated with oTR (1 μM) for 24 h. The protein levels were detected by western blot analysis. The experiments were repeated three times and the data show representative results.

It has been reported that SHP-1 and SHP-2 have important roles in hematopoietic cells [18,19]. The expression of SHP-1 in HL-60 cells was examined. We found that oTR increased phosphorylated SHP-1 protein expression (Figure 6). SHP-2 participates in JAK/STAT signaling and positively contributes to cell differentiation and cell cycle maintenance [20,21,22]. The effect of oTR on the expression of phosphorylated SHP-2 was also detected. Here we found that oTR inhibited the phosphorylation of SHP-2.

## Discussion

Differentiation therapies involve conversion of malignant tumors to curable tumors or terminally differentiated cells that undergo no further proliferation [23].

AML has been classified into eight subtypes, M0 to M7, according to the French-American-British group, which used morphology and cytochemistry to characterize AML [24,25]. Acute promyelocytic leukemia (APL) is the AML-M3 subtype characterized by the t(15;17) translocation [26]. ATRA and ATO are therapeutics specifically designed for this molecular feature [27]. Although these two drugs have greatly improved the prognosis for APL patients, they are not suitable for other subtypes of AML. Therefore, new differentiation-induced agents are needed for AML.

The HL-60 cell line is the M2 subtype of AML [28]. In the present study, we found that cytokinin oTR was effective for inducing granulocytic differentiation of HL-60 AML cells. oTR increased the phosphorylation of SHP-1 while inhibiting the phosphorylation of SHP-2. We also found that oTR reduced the expression of phosphorylated STAT3 and the upstream kinase, Janus kinase 2, in a time-dependent manner. Our findings indicate that oTR can exert antitumor activity in HL-60 cells by inducing differentiation through the STAT3 signaling pathway.

Cytokinins are a class of naturally occurring plant hormones and purine derivatives that play important roles in plant growth and differentiation; moreover, they exhibit anticancer activity in vitro and in vivo in mammals [12,14]. Thus, cytokinins may be useful in the treatment of human diseases that involve dysregulated cell proliferation and/or differentiation [12].

oTR is a naturally occurring nucleoside that can be extracted from plants. In our study, we showed that oTR significantly inhibited the proliferation of HL-60 AML cells, as indicated by reduced viability of HL-60 cells upon treatment with oTR. Furthermore, we found that HL-60 cells were induced into mature granulocytes when treated with oTR. oTR also arrested the cell cycle at the S phase and upregulated the expression of CD11b. We confirmed the induction of differentiation by morphological analysis. We found that treatment with oTR reduced the nuclear cytoplasmic ratio and altered the horseshoe morphology of nuclei. Taken together, these findings indicated that oTR induced the differentiation of HL-60 cells into mature granulocytes.

STAT1 and STAT3 activation is important for the terminal differentiation of immature leukemia cells [29]. The activation of STAT1 has been confirmed in the differentiation of various drug-induced leukemia cells [30,31,32]. Activation of STAT1 is important in ATRA and other various drug-induced differentiation therapies for myeloid cells in APL and other subtypes of acute leukemia. Phosphorylated STAT1 can transactivate downstream target genes, such as *PU.1, C/EBP*α*, C/EBP*β*, CXCL-10, RIG-G*, and* IRF-I*, in order to induce cell differentiation.

Abnormal STAT3 activation is often detected in many human cancer cells, including leukemia [33]. Homodimerization of STAT3 can lead to nuclear translocation, DNA binding, and subsequent gene transcription involved in the activation of STAT3 [33]. During activation, STAT3 phosphorylation is performed through activation of its upstream Janus kinases [34]. Agents that suppress the activation of STAT3 reportedly have potential for cancer prevention and treatment [6]. Here, we found that oTR suppressed STAT3 activation in oTR-induced AML differentiation therapy, while it induced the activation of STAT1; moreover, oTR induced the regulation of transcription factors C/EBPα, C/EBPβ, and PU.1, all of which are important during myeloid differentiation.

SHP-1 is encoded by the *PTPN6* gene and expressed widely in the hematopoietic system; it exhibits various impacts on cell signaling pathways [6,35]. SHP-1 has been reported to negatively regulate the phosphorylation of STAT3 during tumor development, including in the formation of leukemias, as well as in gastric and breast cancers [20,36]. SHP-2 is encoded by the *PTPN11* gene; its overexpression has been observed at both protein and RNA levels in human AML cell lines [37]. Notably, SHP-2 can inhibit apoptosis in cancer stem cells and enhance the growth of leukemia stem cells [20,37,38]. Although SHP-2 is traditionally regarded as a PTP, such that it should inhibit the activity of kinases and exhibit negative regulation of cell function, SHP-2 has been reported to promote cell growth through both upregulation of positive signaling pathways and downregulation of negative signaling pathways [39,40,41]. Here, we found that oTR inhibited the phosphorylation of SHP-2, whereas it increased the phosphorylation of SHP-1 in HL-60 cells.

## Conclusion

Our results demonstrated that oTR induced the differentiation of HL-60 human AML cells by suppression of the STAT3 signaling pathway and induction of STAT1 activation. We also found that oTR reduced the level of phosphorylated SHP-2, while it increased the level of phosphorylated SHP-1 in HL-60 cells. Our data suggest that oTR might be applicable in the treatment of AML patients with M2 subtype by inducing cell differentiation, but not all subtypes of AML. Therefore, future studies on the antitumor effect of oTR are needed.

## Figures and Tables

**Table 1:**
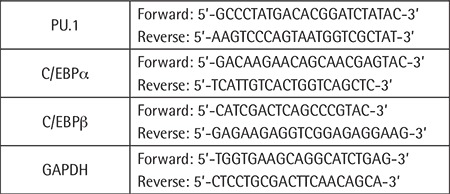
The sequences of primers for real-time polymerase chain reaction.
